# Supplementary material for: Fully Automated Production of [68Ga]GaFAPI-46 with Gallium-68 from Cyclotron Using Liquid Targets
Source: Int J Mol Sci. 2023 Oct 12;24(20):15101. doi: 10.3390/ijms242015101 (PMC10606668; doi:10.3390/ijms242015101)
Supplement: Supplementary file 1 [file ijms-24-15101-s001.zip › ijms-2629690-supplementary.pdf]

Table S1. Repeatability results for (a) [^{68}Ga]GaFAPI-46 and (b) [^{nat}Ga]GaFAPI-46.

(a)

Injection	Acitvity (MBq/mL)	Inj. Time	Δt (min)	Corrected Activity	Peak Area	RF [1]
[^{68}Ga]GaFAPI-46	46,88	20:48	96	0,3524	89014,3	252595,5
[^{68}Ga]GaFAPI-46		21:05	113	0,2963	78107,2	263581,1
[^{68}Ga]GaFAPI-46		21:21	129	0,2517	69723,4	276969,8
[^{68}Ga]GaFAPI-46		21:37	145	0,2139	55882,1	261310,9
[^{68}Ga]GaFAPI-46		21:54	162	0,1795	43717,5	243106,8
[^{68}Ga]GaFAPI-46		22:11	179	0,1512	41455,1	274143,3
**%RSD**						**4,89**

(b)

Injection	Area
[^{nat}Ga]GaFAPI-46	97,59
[^{nat}Ga]GaFAPI-46	104,38
[^{nat}Ga]GaFAPI-46	101,67
[^{nat}Ga]GaFAPI-46	103,79
[^{nat}Ga]GaFAPI-46	106,10
[^{nat}Ga]GaFAPI-46	107,52
**%RSD**	**3,40**

[1] $\mathrm{RF} = \frac{\text{Peak area}}{\text{Radioactivity injection time}}$

Table S2. Repeatability results of radioTLC method for [^{68}Ga]GaFAPI-46.

Injection	Area of [^{68}Ga]GaFAPI-46 (%)
[^{68}Ga]GaFAPI-46	99.94
[^{68}Ga]GaFAPI-46	99.91
[^{68}Ga]GaFAPI-46	99.78
[^{68}Ga]GaFAPI-46	99.88
[^{68}Ga]GaFAPI-46	99.94
[^{68}Ga]GaFAPI-46	99.63
**%RSD**	**0.12**

Table S3. Repeatability results of radioTLC method for ethanol content in [^{68}Ga]GaFAPI-46.

Injection	Area of [^{68}Ga]GaFAPI-46 (%)
[^{68}Ga]GaFAPI-46	2491.00
[^{68}Ga]GaFAPI-46	2341.50
[^{68}Ga]GaFAPI-46	2529.10
[^{68}Ga]GaFAPI-46	2589.40
[^{68}Ga]GaFAPI-46	2594.40
[^{68}Ga]GaFAPI-46	2589.30
**%RSD**	**3.88**

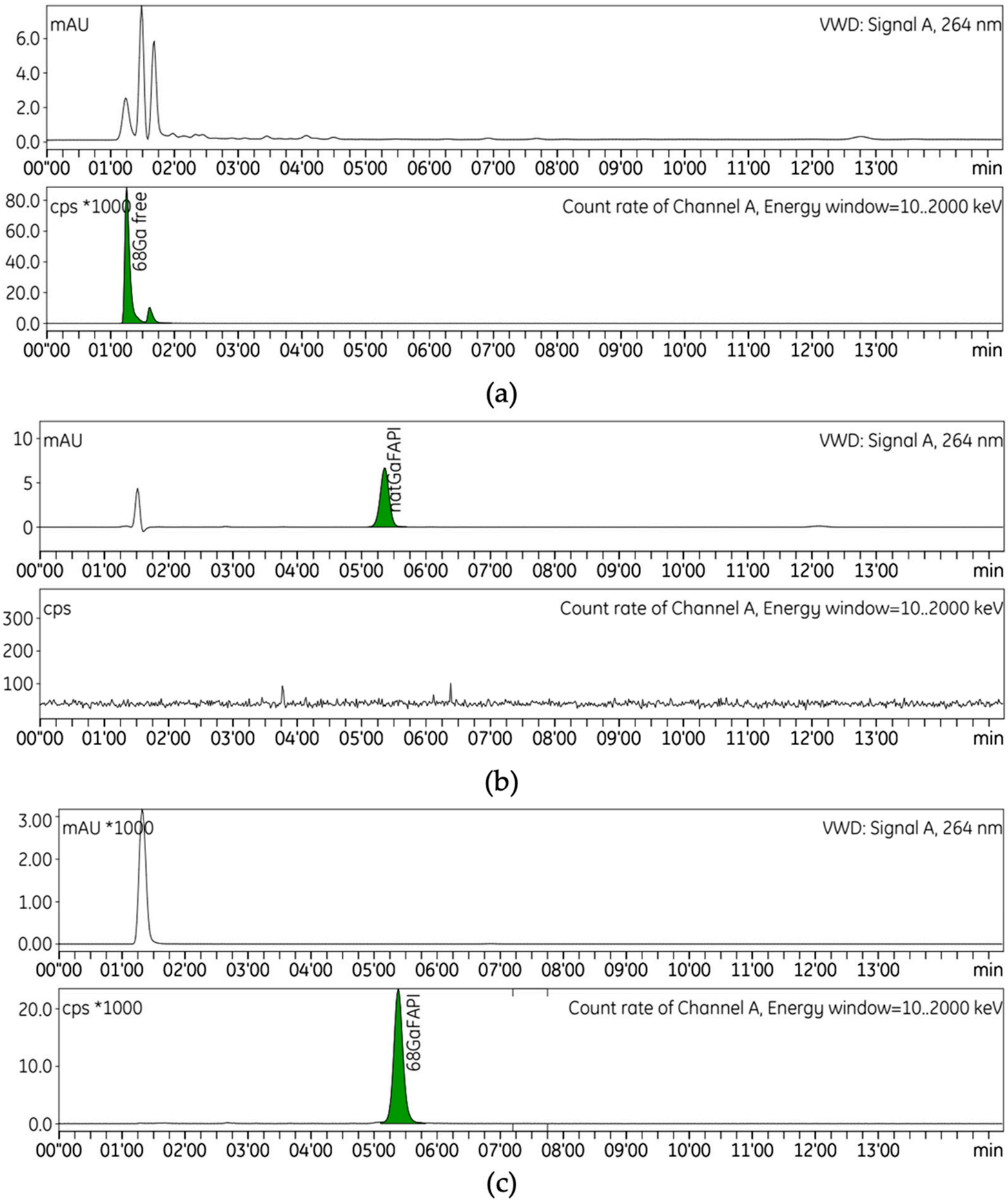

Figure S1. Representative HPLC chromatograms of each solution: (a) [^{68}Ga]$GaCl_3$, (b) [^{nat}Ga]GaFAPI-46, and (c) [^{68}Ga]GaFAPI-46.

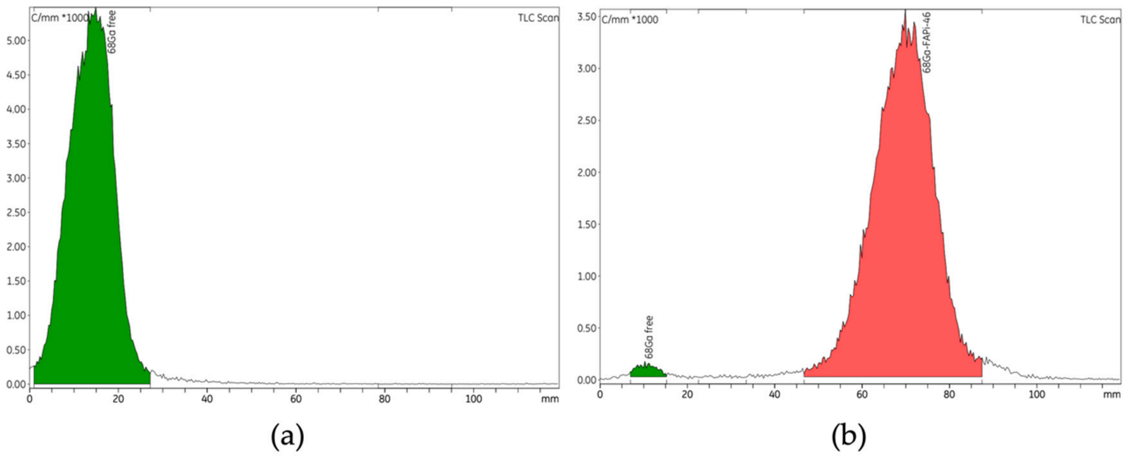

Figure S2. Representative radioTLC chromatograms of each solution: (a) [^{68}Ga]$GaCl_3$ and (b) [^{68}Ga]GaFAPI-46.

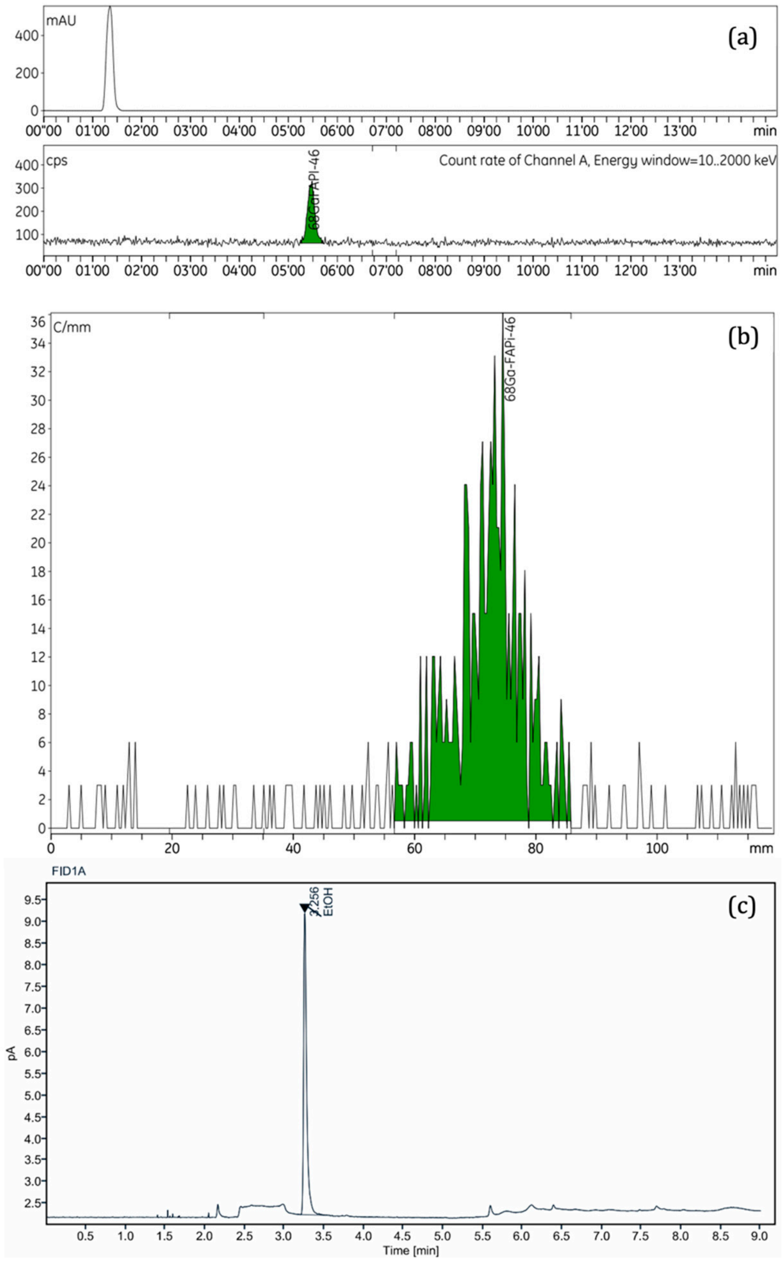

Figure S3. LOQ Representative chromatograms of HPLC, TLC and GC methods.

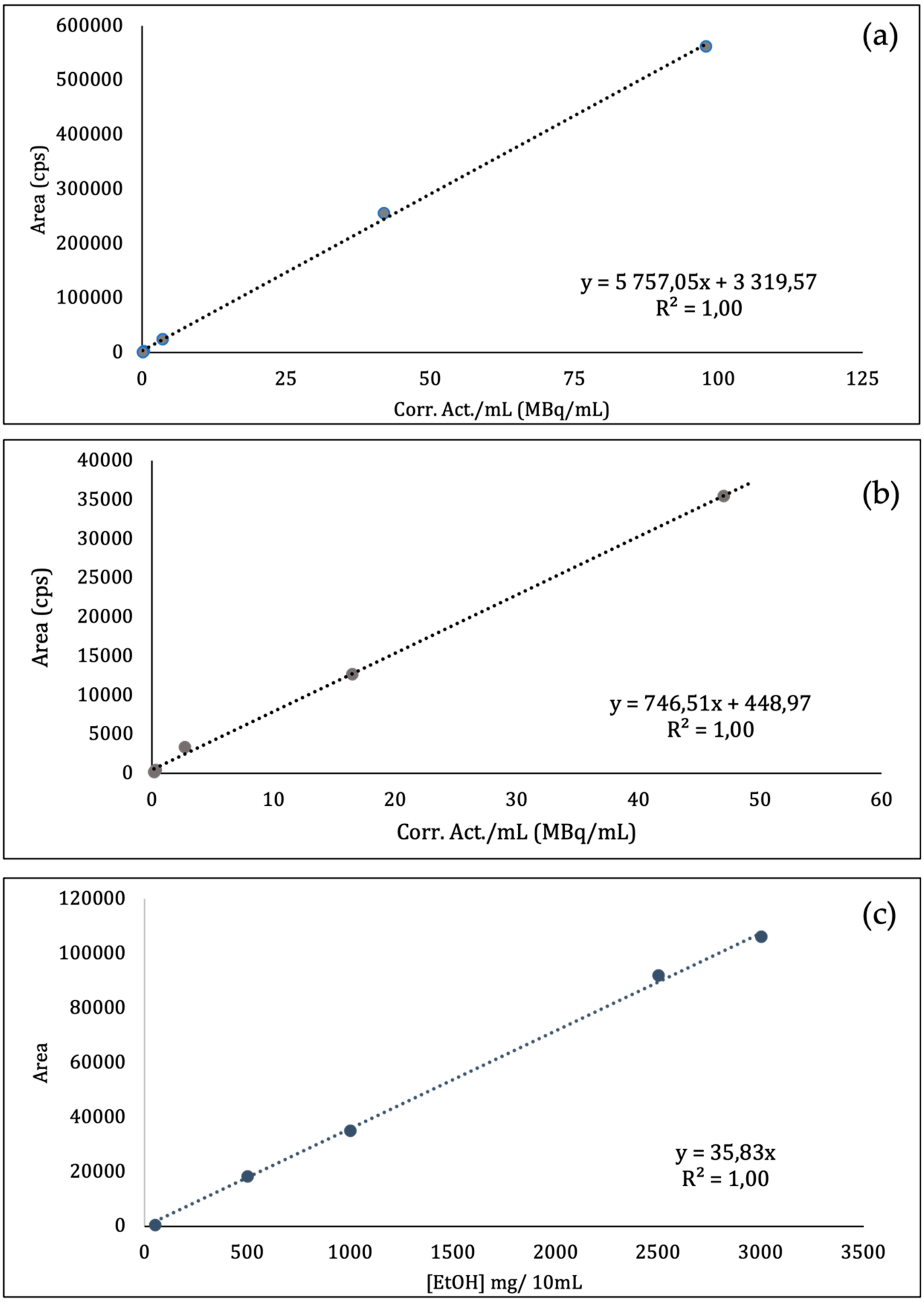

Figure S4. Linearity plot of (a) HPLC, (B) TLC and (C) GC methods.
